# Comparative analysis of the mitochondrial genomes of oriental spittlebug trible Cosmoscartini: insights into the relationships among closely related taxa

**DOI:** 10.1186/s12864-018-5365-7

**Published:** 2018-12-27

**Authors:** Tianjuan Su, Bo He, Kui Li, Aiping Liang

**Affiliations:** 1 Key Laboratory of Zoological Systematics and Evolution, Institute of Zoology, Chinese Academy of Sciences, Beijing, 100101 China; 2 College of Life Sciences, University of Chinese Academy of Sciences, Beijing, 100049 China; 3 Key Laboratory of Cultivation and Protection for Non-Wood Forest Trees, Ministry of Education, Central South University of Forestry and Technology, Changsha, 410004 China

**Keywords:** Mitochondrial genome, Spittlebug, Cosmoscartini, Phylogeny

## Abstract

**Background:**

Cosmoscartini (Hemiptera: Cercopoidea: Cercopidae) is a large and brightly colored Old World tropical tribe, currently containing over 310 phytophagous species (including some economically important pests of eucalyptus in China) in approximately 17 genera. However, very limited information of Cosmoscartini is available except for some scattered taxonomic studies. Even less is known about its phylogenetic relationship, especially among closely related genera or species. In this study, the detailed comparative genomic and phylogenetic analyses were performed on nine newly sequenced mitochondrial genomes (mitogenomes) of Cosmoscartini, with the purpose of exploring the taxonomic status of the previously defined genus *Okiscarta* and some closely related species within the genus *Cosmoscarta*.

**Results:**

Mitogenomes of Cosmoscartini display similar genomic characters in terms of gene arrangement, nucleotide composition, codon usage and overlapping regions. However, there are also many differences in intergenic spacers, mismatches of tRNAs, and the control region. Additionally, the secondary structures of rRNAs within Cercopidae are inferred for the first time.

Based on comparative genomic (especially for the substitution pattern of tRNA secondary structure) and phylogenetic analyses, the representative species of *Okiscarta uchidae* possesses similar structures with other *Cosmoscarta* species and is placed consistently in *Cosmoscarta*. Although *Cosmoscarta bimacula* is difficult to be distinguished from *Cosmoscarta bispecularis* by traditional morphological methods, evidence from mitogenomes highly support the relationships of (*C. bimacula* + *Cosmoscarta rubroscutellata*) + (*C. bispecularis* + *Cosmoscarta* sp.).

**Conclusions:**

This study presents mitogenomes of nine Cosmoscartini species and represents the first detailed comparative genomic and phylogenetic analyses within Cercopidae. It is indicated that knowledge of mitogenomes can be effectively used to resolve phylogenetic relationships at low taxonomic levels. Sequencing more mitogenomes at various taxonomic levels will also improve our understanding of mitogenomic evolution and phylogeny in Cercopidae.

**Electronic supplementary material:**

The online version of this article (10.1186/s12864-018-5365-7) contains supplementary material, which is available to authorized users.

## Background

The mitochondrial genome (mitogenome) of most metazoan is circular and compact with relatively conserved gene organization, order and direction. It varies from 14 to 20 kb and contains 13 protein-coding genes (PCGs), two ribosomal RNA genes (rRNAs), 22 transfer RNA genes (tRNAs), and a large non-coding region (also referred to as the control region) [1–3]. Owing to some unique features like small size, high copy numbers, maternal inheritance, strict orthologous genes, low rate of recombination, and accelerated rate of nucleotide substitution [4, 5], the mitogenome has been extensively used in various study areas, including species identification, population genetics, phylogeny and evolution [6–9]. Genome-level characters, including nucleotide composition, structural genomic features, and gene rearrangement, have also been widely used for comparative and evolutionary genomics, and phylogenetic inference at different taxonomic levels [7, 10, 11]. TRNAs with different patterns in base composition, gene rearrangement, and secondary structure are proposed as useful ways to study the evolution of mitogenomes and can be used as powerful phylogenetic markers [12–15]. Furthermore, mitogenomes, especially in intergenic spaces, usually present higher mutation rates than nuclear genes and may provide valuable information for phylogenetic analyses among closely related taxa [10, 16, 17].

Cosmoscartini (Hemiptera: Cercopoidea: Cercopidae) is a large and brightly colored Old World tropical tribe, including more than 310 described species in approximately 17 genera [18–20]. Just as other spittlebugs, during the nymphal period, they present the habit of producing copious spittle masses to cover themselves inside through continuously sucking the liquid and nutrients contained in xylem tissue, which may cause serious economic damage to host plants [21, 22]. In terms of ecological function, spittle mass has traditionally been seen as an effective barrier against predation, parasitism, and desiccation [21, 23]. In addition, the spittle mass functions as a light attenuator, which could reduce potentially damaging solar radiation [24]. Furthermore, nymphs of many spittlebug species are known to aggregate in one spittle mass and inflict more serious economic damage [25]. Some species of *Cosmoscarta* are reported as economically important pests of eucalyptus in China [26]. Therefore, it is very important to accurately elucidate the taxonomic status and phylogenetic relationships of Cosmoscartini species. However, only very limited studies about Cosmoscartini are available except for some scattered taxonomic studies [27, 28] and the detailed biology of Cosmoscartini species is likewise scattered. Additionally, some taxa are difficult to identify to species due to a lack of sufficient diagnostic characters to illustrate distinctions [21]. Even less is understood about the phylogenetic relationship within Cosmoscartini, especially among closely related genera or species. There is a controversy related to whether or not *Okiscarta* should be raised to genus level [18–20]. Therefore, detailed molecular data is required for a comprehensive phylogenetic analysis of the tribe Cosmoscartini.

To date, only one complete mitogenome of Cosmoscartini has been sequenced (GenBank accession number KP064511), which is quite limited and restricts our understanding of the phylogeny of Cosmoscartini. In the present study, we sequenced mitogenomes of nine Cosmoscartini species, including one *Ectemnonotum*, one *Okiscarta* (this genus has only two species totally), and seven *Cosmoscarta* species, respectively. The aims of this study were to: 1) provide a detailed comparative analysis of these mitogenomes, including nucleotide composition, codon usage, secondary structures of RNAs, and novel features of the control region; 2) investigate the phylogenetic relationships among three genera of Cosmoscartini, especially for the taxonomic status of the previously defined genus *Okiscarta*; 3) explore the relationships among some closely related species, which were difficult to distinguish from traditional morphological methods.

## Results

### Genome structure

**Fig. 1:**
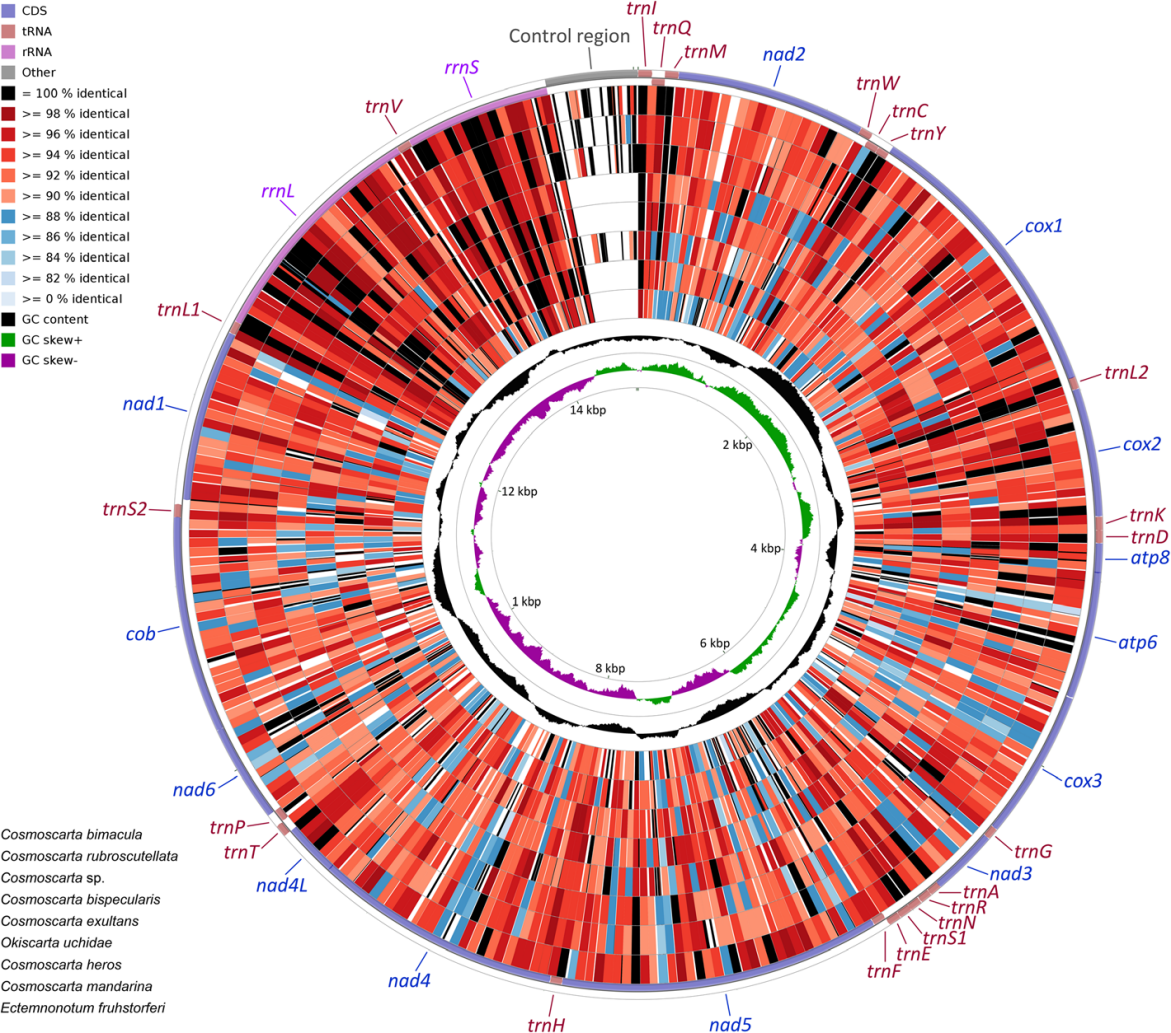
Circular diagram of the nine Cosmoscartini mitogenomes. Different color is performed to show the nucleotide identity of BLAST hits, with the reference genome represented by *C. bimacula*. The rings are arranged in an order that the most similar genome is placed closest to the outer edge of the circle

This study presented five complete and four nearly complete mitogenomes with the absence of the control region (Fig. 1). The total length of each complete mitogenome ranged from 15,024 bp in *Cosmoscarta bimacula* to 15,677 bp in *Cosmoscarta rubroscutellata* (Additional file 1: Table S1). The sequenced mitogenomes contained the entire set of 37 genes. The J-strand carried most of the genes (9 PCGs and 14 tRNAs), while the remaining genes (4 PCGs, 8 tRNAs and two rRNAs) were located on the N-strand (Fig. 1). Gene arrangement was identical within Cosmoscartini and was consistent with the putative ancestral type of insects [2, 7]. All the sequenced genomes were relatively compact with genes overlapping at 12–15 locations, in which the longest overlap (8 bp) was between *trnW* and *trnC*. These overlapping sequences were highly conserved in length among all sequenced Cosmoscartini mitogenomes. Two PCG pairs, including *atp8-atp6* and *nad4-nad4l*, both overlapped 7 bp and shared the similar sequence (ATGNTAA), which had also been reported in many other insect mitogenomes [29, 30]. There were also 5–7 intergenic spacers (in addition to the control region), with the longest intergenic spacer located between *trnS2* and *nad1*. In addition, 14–19 pairs of genes were directly adjacent with one another including the pairs of *rrnL-trnV*, *trnV-rrnS*, *rrnS-*CR, and CR*-trnI*.

To better visualize the sequence identity in Cosmoscartini mitogenomes, the comparable circular diagram was generated (Fig. 1). Pairwise comparisons between *C. bimacula* and other Cosmoscartini species revealed an overall similarity from 86% (*C. bimacula* vs. *Ectemnonotum fruhstorferi*) to 92% (*C. bimacula* vs. *C. rubroscutellata*). The *rrnL*, *rrnS*, and some tRNA genes (e.g., *trnI*, *trnL1*, and *trnL2*) exhibited much higher levels of sequence conservation. Conversely, control region was the most variable region. Within the PCGs, cytochrome oxidase genes were more conserved with *cox1* and *cox2* presented the highest conservation, whereas NADH dehydrogenase subunit genes were more variable with *nad3* showed maximal variation.

### Nucleotide composition and codon usage

**Fig. 2:**
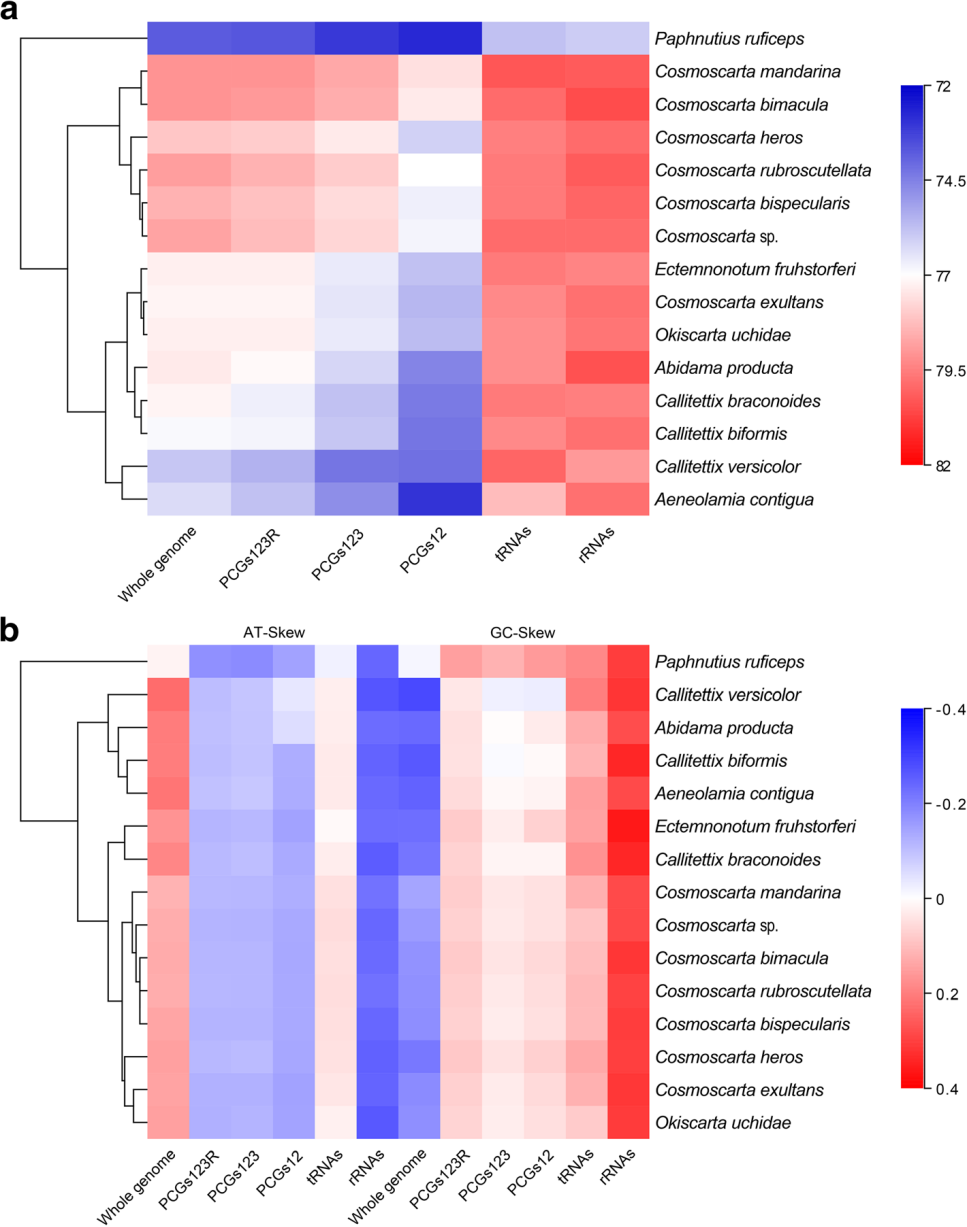
Base composition of various datasets among Cercopidae mitogenomes. (**a**) Hierarchical clustering of Cercopidae species (y-axis) based on their A + T content; (**b**) Clustering of species in terms of AT-skew and GC-skew

The nucleotide composition was observed to be similar among the five complete Cosmoscartini species, with the overall A + T content ranging from 78.1% in *Cosmoscarta heros* to 79.1% in *C. bimacula*. These results confirmed an AT-bias, which were also higher than other Cercopidae species (73.8–77.4%) (Fig. 2a; Additional file 1: Table S1). The segment with highest A + T content was found in control region (80.5–84.8%), which was generally higher than other Cercopidae species (74.5–82.5%). The lowest A + T content within Cosmoscartini was found in PCGs (76.5–78.7%), which was also higher than other Cercopidae species (73.1–76.2%). The biased usage of A + T nucleotides was also reflected in codon frequencies. Relative synonymous codon usage (RSCU) of Cosmoscartini revealed that degenerate codons were biased to use more A/T than G/C in the third codon position (Additional file 2: Table S2). Some GC-rich codons were seldom utilized in the Cosmoscartini species, such as GCG and CGC which were absent in six and four of all nine species, respectively. Conversely, the four most prevalent codons in Cosmoscartini, including TTT (*trnF*), TTA (*trnL2*), ATT (*trnI*), and ATA (*trnM*), were all composed of A and/or T. AT-skews (from 0.119 to 0.169) and GC-skews (from − 0.227 to − 0.141) in Cosmoscartini mitogenomes were similar to patterns typically found in other Cercopidae species, i.e., positive AT-skew and negative GC-skew for the J-strand (Fig. 2b).

### Protein-coding genes

**Fig. 3:**
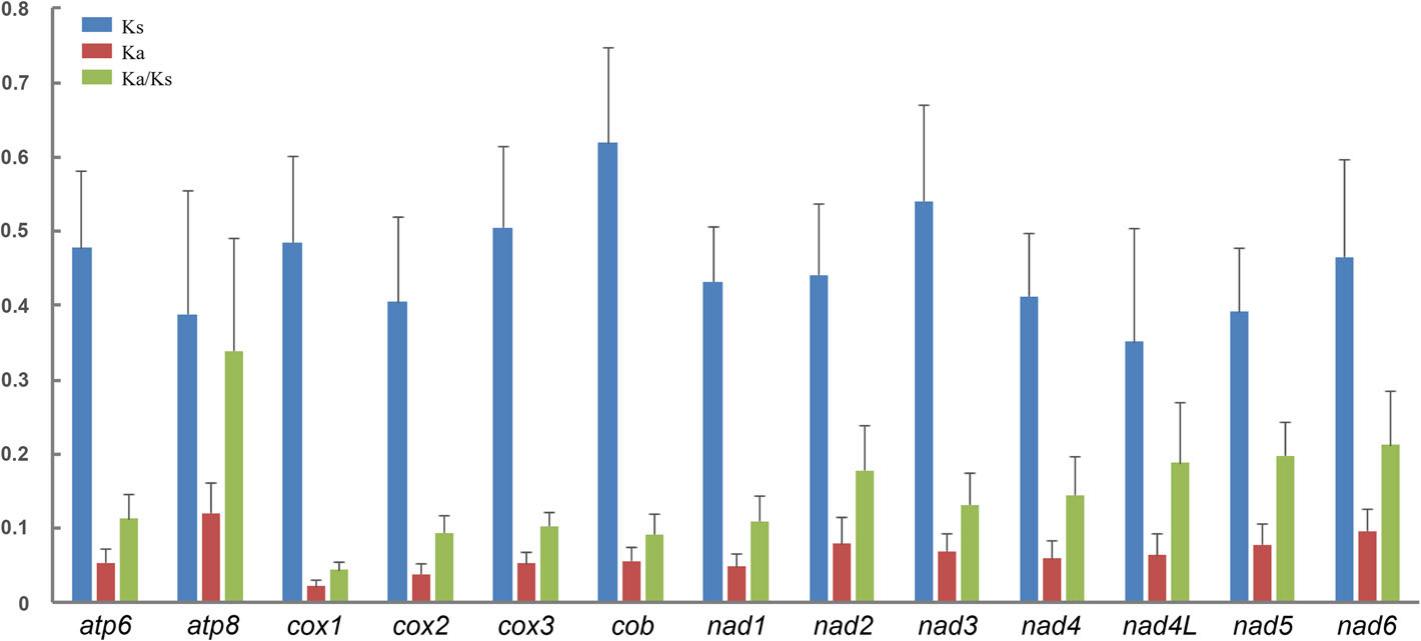
Evolutionary rate of each PCG among Cosmoscartini species. Ks, synonymous nucleotide substitutions per synonymous site; Ka, nonsynonymous nucleotide substitutions per nonsynonymous site

Orthologs from the Cosmoscartini mitogenomes had similar start and stop codons (Additional file 3: Table S3). Most PCGs exhibited the typical start codon ATN, but *cox1*, *nad2*, and *nad5* in all the species initiated with TTG. While most PCGs ended with the termination codon TAA or TAG, truncated codon T was also detected in *cox2*, *nad4*, and *nad5* in the tribe. Truncated stop codons were common in insect mitogenomes and might be completed by post-transcriptional polyadenylation [31]. In order to assess the evolutionary patterns of PCGs, the values of Ka, Ks, and Ka/Ks were calculated (Fig. 3), with *atp8* and *cox1* presented the highest and lowest evolutionary rate, respectively. In addition, the Ka/Ks ratio for each PCG was far lower than 1.

### Comparison of tRNA secondary structures

**Fig. 4:**
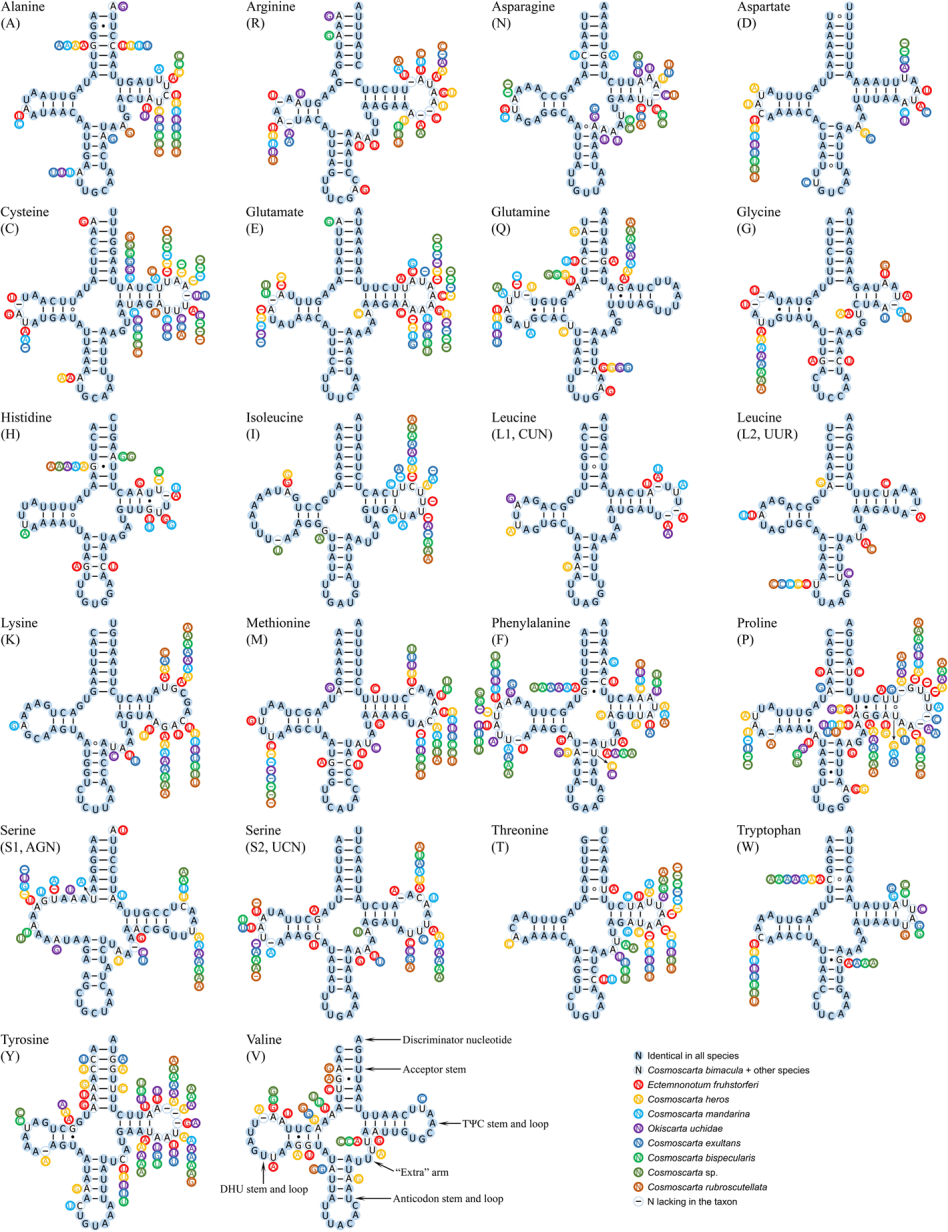
Secondary structures of tRNA families in Cosmoscartini mitogenomes. The nucleotide substitution pattern for each tRNA is modeled using as reference the structure predicted for *C. bimacula*. Watson-Crick base pairings, GU bonds, and mismatches are illustrated by dashes, solid dots, and hollowed dots, respectively

All 22 tRNAs typical of mitogenomes of bilateral animals were found in the nine Cosmoscartini mitogenomes (Fig. 4). Most tRNAs could be folded into the canonical cloverleaf structure except for *trnS1*, with its dihydrouracil (DHU) arm forming a simple loop, which was considered a typical feature in metazoan mitogenomes [32]. Moreover, we found that *trnS1* in all nine Cosmoscartini mitogenomes had an unusual anticodon stem, with an unpaired nucleotide. Similar pattern was also found in the *trnR* acceptor stem. This was an unusual phenomenon, but had also been reported in the anticodon and acceptor stems of some other hemipterans [33, 34].

Except for the most conserved region of anticodon arm, the conservation of each stem was always higher than the corresponding loop. Conversely, the TΨC loop was the most variable structure, followed by the DHU loop and “extra” arm as the second and third most variable, respectively. According to the substitution patterns proposed by Negrisolo et al. [35], two patterns were summarized: (1) fully compensatory base changes (cbcs) (e.g., G-C vs. A-U); (2) hemi-cbcs (e.g., G-U vs. A-U). All the stem-base changes presented in Cosmoscartini tRNAs could be explained by these two patterns, except for several potential mismatches (e.g. A-A vs. C-A in the acceptor stem of *trnW*). However, the nucleotide substitutions on loops could not be modeled clearly because of a high level of variation. In some conserved tRNAs (e.g., *trnI* and *trnD*), nucleotide substitutions were generally restricted to TΨC and DHU loops and extra arms (Fig. 4), with changes on stems reduced to only 0–1 fully cbcs (e.g., G-C vs. A-T in the TΨC stem of *trnI*).

### Comparison of rRNA secondary structures

**Fig. 5:**
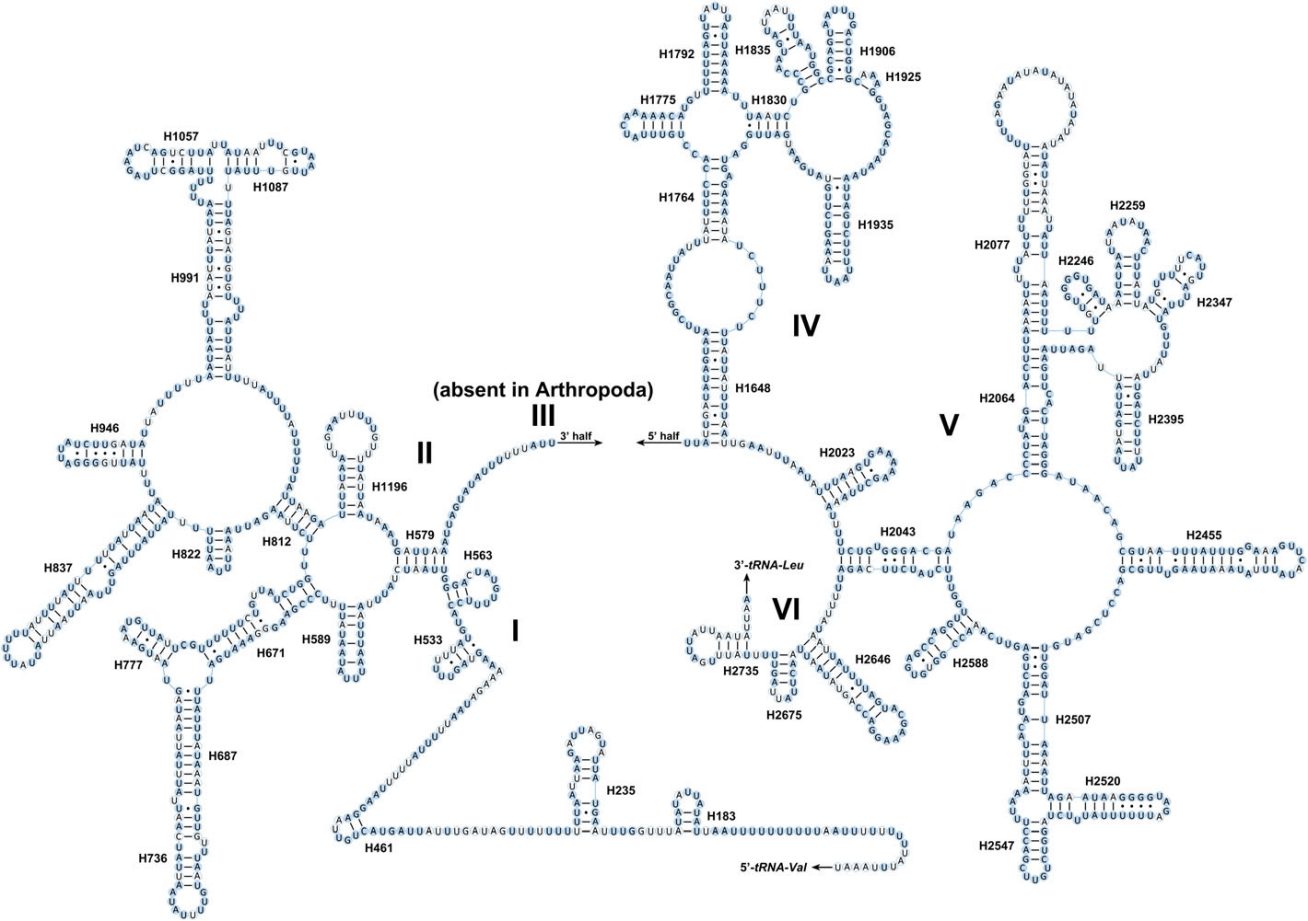
Predicted secondary structure of the *rrnL* in the mitogenome of *C. bimacula*. Filled circle, nucleotide conserved in nine Cosmoscartini mitogenomes; hollowed circle, nucleotide not conserved. Roman numerals denote the conserved domain structure. Watson-Crick pairs are connected by dashes, whereas GT pairs are joined by dots

The secondary structure of *rrnL* consisted of 44 helixes in five domains (I-II, IV-VI) of insect *rrnL* that do not have domain III [36]. The multiple alignment of Cosmoscartini *rrnL* extended over 1258 positions and contained 969 conserved (77.03%) and 289 variable sites (22.97%), respectively. Conserved nucleotides were distributed unevenly, with domains IV and V more conserved than domains I, II, and VI (Fig. 5).

**Fig. 6:**
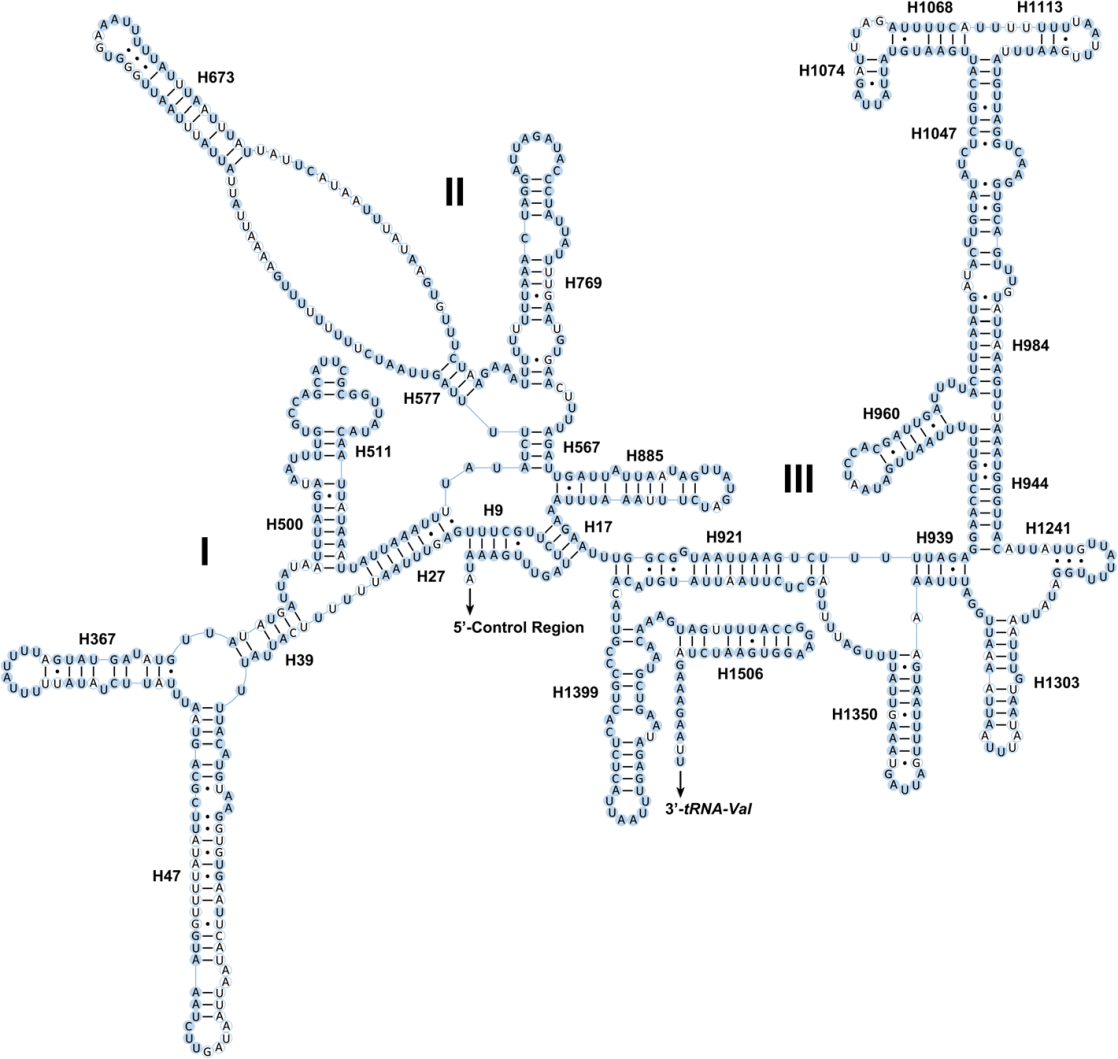
Predicted secondary structure of the *rrnS* in the mitogenome of *C. bimacula*. Filled circle, nucleotide conserved in nine Cosmoscartini mitogenomes; hollowed circle, nucleotide not conserved. Roman numerals denote the conserved domain structure. Watson-Crick pairs are connected by dashes, whereas GT pairs are joined by dots

The *rrnS* contained three domains and 27 helixes, with a limited number of non-canonical pairings (e.g., G-A on helix H944). The multiple alignments of Cosmoscartini *rrnS* spanned 776 positions and contained 633 conserved (81.57%) and 143 variable (18.43%) positions, respectively. Nucleotide conservation among domains and helixes was distributed unevenly (Fig. 6). Compared to domain I and II, domain III was structurally more conserved within Cosmoscartini.

### Non-coding regions

Except for the control region, Cosmoscartini mitogenomes were highly economized in size with only 5–7 intergenic spacers, most of which were shorter than 5 bp. However, the longest intergenic spacer was found between *trnS2* and *nad1* (17–26 bp) in all Cosmoscartini mitogenomes (Additional file 4: Figure S1), sharing a conserved sequence of AAC(C/T)A(A/C)AAA(T/C)AATGAA.

Overall, Cosmoscartini control regions presented distinct sequence and structure features, such as size variation and different tandem repetitions (Additional file 5: Figure S2). The repeat units ranged from 15 to 355 bp, and the copy number ranged from 1.8 to 4.8. Most of the length variation of control regions among species was due to the variable lengths of tandem repeats, such as the control region (1135 bp in total) of *Cosmoscarta* sp., the majority of which was made up of a 721 bp tandem-repeat region, containing 2 large tandem duplications with the high similarity of 98%.

### Phylogenetic analysis

**Fig. 7:**
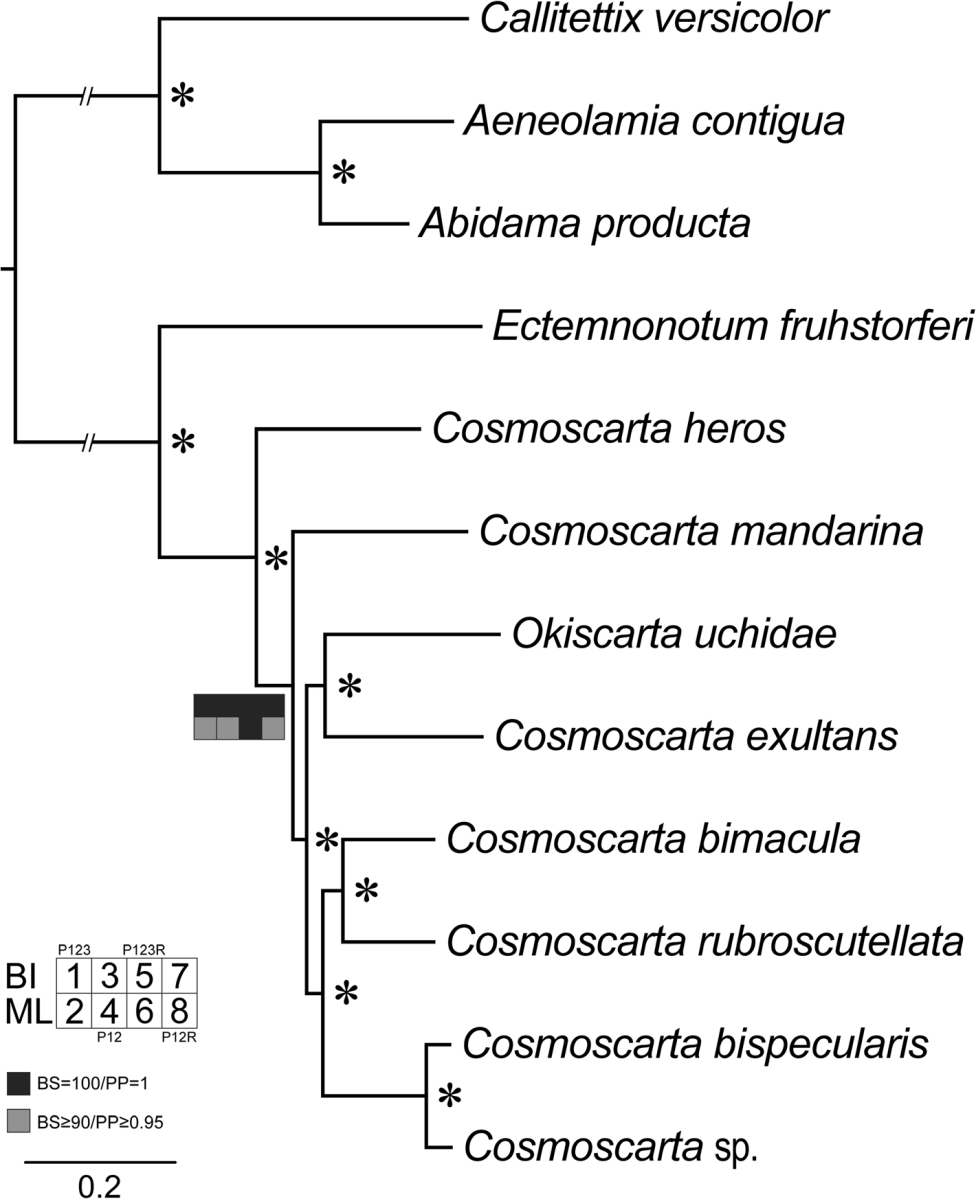
Phylogenetic tree inferred from mitogenomes of Cosmoscartini. Squares at the nodes are Bayesian posterior probabilities (PP) for 1, 3, 5, and 7, Bootstrap values (BS) for 2, 4, 6, and 8. Dataset of P123, 1 and 2; P12, 3 and 4; P123R, 5 and 6; P12R, 7 and 8. * indicates PP = 1.00 and BS = 100 in all inferences

The tree topologies obtained from all BI and ML analyses were identical (Fig. 7). The monophyly of Cosmoscartini was highly supported, with genus *Ectemnonotum* (represented by the species *E. fruhstorferi*) inferred as sister to other Cosmoscartini species (BI = 1.0 and ML = 100). However, *Cosmoscarta* was recovered as a non-monophyletic group in all analyses, with respect to the previously mentioned *Okiscarta uchidae*,

To further investigate the phylogenetic results described above, the AU, KH, and SH tests for constrained topologies were performed (Table 1). The genus *Cosmoscarta* was paraphyletic in the topology of Fig. 7. The alternative phylogeny implying its monophyly is rejected in all topology tests (*P* < 0.05).

**Table 1:** Alternative tree topologies tests for the genus *Cosmoscarta*

Datasets	Hypothesis	-Ln likelihood	AU	KH	SH
P123	Non-monophyly	−50,660.019	0.993	0.995	1.000
	Monophyly	−50,690.365	**0.007**	**0.005**	**0.002**
P12	Non-monophyly	−25,942.714	0.993	0.995	1.000
	Monophyly	−25,975.926	**0.008**	**0.005**	**0.005**
P123R	Non-monophyly	−61,049.402	1.000	1.000	1.000
	Monophyly	−61,096.447	**0.000**	**0.000**	**0.001**
P12R	Non-monophyly	−36,323.658	1.000	0.999	1.000
	Monophyly	−36,372.380	**0.000**	**0.001**	**0.001**

*Note*: The significant values (*P* < 0.05) are in boldface, indicating that the monophyly is rejected

Within *Cosmoscarta*, although *C. bimacula* is difficult to be distinguished from *Cosmoscarta bispecularis* due to their highly similar morphology, phylogenetic analyses based on mitogenome data robustly supported the relationships of (*C. bimacula* + *C. rubroscutellata*) + (*C. bispecularis* + *Cosmoscarta* sp.). From the aspect of comparative mitogenomics analysis, *C. bimacula* also had the highest nucleotide similarity with *C. rubroscutellata*. *C. bispecularis* and *Cosmoscarta* sp. not only shared the identical length in each PCG, but also had similar substitution patterns in the secondary structures of tRNAs.

## Discussion

This study presents nine mitogenomes of Cosmoscartini,in which five species were completely sequenced. However, due to the high A + T content and complicated secondary structure, we were unable to amplify control regions in the four other species. Among the five complete mitogenomes, the control region also exhibited more variation in length (varying from 484 bp in *C. bimacula* to 1152 bp in *C. rubroscutellata*) in comparison to the other regions. Furthermore, although stem-loop structures could be found at the 3′-end of the control regions in all the complete Cosmoscartini species, no obvious conserved functional motifs could be detected. Additionally, two microsatellite-like repeat regions were found in *C. bispecularis*, but not in the other species. These results showed that the control region was a fast-evolving sequence with some taxon-specific characteristics, which might be valuable genetic markers for evolutionary and population genetic studies within species groups. Among all nine newly sequenced mitogenomes, each PCG was found to be the same in length, except for *cob*, where *C. bispecularis* and *Cosmoscarta* sp. sharing the same length (1137 bp) but having 3 nucleotides more than that of other species (1134 bp). Because of stable secondary structures, there was also limited length variation in all tRNAs among different species, ranging from 1467 bp in *C. bispecularis* to 1478 bp in *O. uchidae*. The rRNAs also shared the generally consistent lengths, ranging from 2015 bp in *Cosmoscarta* sp. to 2034 bp in *C. bimacula*.

It was indicated that the evolutionary patterns of PCGs were highly consistent among Cosmoscartini and presented some common characters: (1) *atp8* appeared to have the highest evolutionary rate and might be suitable for analyzing intraspecies relationships [37]; (2) *cox1*, which was used as the DNA barcoding marker [38], had the lowest evolutionary rate. Similarly, two other cytochrome oxidase genes (*cox2* and *cox3*) and the cytochrome b gene (*cob*) also had the relatively lower evolutionary rates than the remaining genes, suggesting that they might be potential barcoding markers. (3) The Ka/Ks ratio for each PCG was far lower than 1, indicating that all PCGs were evolving under purifying selection and could be used to investigate phylogenetic relationships within Cosmoscartini.

Based on the comparative secondary structures of tRNAs, nucleotide substitutions on both stems (especially cbcs and hemi-cbcs) and loops characterizing taxa at different taxonomic levels might provide evidence for the phylogenetic analyses. For example, the variable tRNAs of *E. fruhstorferi* were quite different from those of other species (Fig. 4), e.g., A-T presented in the *trnG* anticodon stem of *E. fruhstorferi*, while the *Cosmoscarta* and *Okiscarta* species exhibited the G-C pair. Secondary structures of tRNAs between *C. bispecularis* and *Cosmoscarta* sp. appeared to be generally invariable. These substitution patterns supported the results of phylogenetic analyses that *E. fruhstorferi* was distantly related to other Cosmoscartini species and that *C. bispecularis* was closely related to *Cosmoscarta* sp.

Interestingly, even mismatches in tRNAs, including U-U, A-C, A-A, and C-U pairs, were associated with the Cosmoscartini phylogenetic relationships. For example, U-U in the acceptor stem of *trnL1* was highly consistent among Cosmoscartini; U-U in the acceptor stem of *trnT* was conserved among *Cosmoscarta*, but presented U-A in *E. fruhstorferi*; A-A in the acceptor stem of *trnW* was conserved among seven of nine Cosmoscartini species, but presented as C-A in *C. bimacula and C. rubroscutellata*. These mismatches in tRNA stems were common in arthropod mitogenomes and could be restored by post-transcriptional editing processes [39] or represented unusual pairings [36].

The double-helix stems, which play a key role in the formation of secondary structures of tRNAs, might experience strong evolutionary constraints [40]. Different substitution patterns in the stems of tRNAs were associated with different evolutionary history, and thus could provide valuable signals in phylogeny. As shown in Fig. 4, strong heterogeneity was observed between *E. fruhstorferi* (30 individual changes in the stems of 15 tRNAs) and other Cosmoscartini species (0–16 individual changes among different species). However, compared with some of the *Cosmoscarta* species (e.g., *C. heros* and *Cosmoscarta mandarina* exhibited 16 and 9 substitutions, respectively), *O. uchidae* even presented fewer individual nucleotide substitutions (4 individual changes) in the stems of tRNAs. This similar substitution pattern between *O. uchidae* and *Cosmoscarta* indicated that *O. uchidae* shared similar evolutionary history with *Cosmoscarta* species, and might be a member of *Cosmoscarta*.

Conserved nucleotides were distributed unevenly among rRNA genes. For *rrnL*, domains IV and V were more conserved than domains I, II, and VI (Fig. 5). Within domain IV, six helixes (H1775, H1792, H1830, H1906, H1925, and H1935) were highly conserved, with only 0–2 nucleotide substitutions. In domain V, most helixes were conserved, except for helixes H2077 and H2347 which were also highly divergent among other insect mitogenomes [34, 41]. In the variable domains I, II, and VI, except for the helixes of H183, H461, H563, H589, and H822, there were no obvious conserved helixes within Cosmoscartini. For *rrnS*, domain III was structurally more conserved than domain I and II (Fig. 6). The primary exceptions were H1074 and H1113, which had more variable sites in secondary structures. Mfold Web Server predicted several possible helix structures for this complex region because of their high A + T content and several non-canonical base pairs. Nevertheless, according to previously published rRNA structures of hemipterans [34, 42], we chose the one with more conserved nucleotides on the paired stems. Other predicted secondary structures, which presented more variable sites on stems, might be taxon-specific and were not accepted in our analyses. Additionally, although the helix H1068 was absent in some hemipterans [43, 44], it was found in the *rrnS* of Cosmoscartini, as also presented in other insects [45, 46]. In domain I, the helix H47 was highly variable in terms of its sequence and secondary structure. Actually, no consistent structure had been reported for this region in insects [47]. In domain II, although H673 was proposed to form a long stem and a very small loop in some insects [33], this helix formed a relatively short paired stem with a large loop in Cosmoscartini, as reported in other species of Hemiptera [34, 48].

Among the non-coding regions, the spacer of *trnS2-nad1* was supposed to contain the binding site for the bidirectional transcription termination factor (DmTTF) [49]. Furthermore, sequence alignments of this region could also be reflective of the relationships of closely related species. It was indicated that *C. bimacula*, *C. rubroscutellata*, *C. bispecularis*, and *Cosmoscarta* sp. shared the similar length (22–26 bp) and the identical segment of AACCAAAAATAATGAA. *Cosmoscarta exultans* and *O. uchidae* also contained the identical sequence of AACTAAAAATAATGAA. It had been proposed that intergenic region was under relaxed selection and had a high nucleotide substitution rate [50]. However, *O. uchidae* shared the highly conserved nucleotide pattern with other *Cosmoscarta* species and as a result had a close relationship with them or might be a member of *Cosmoscarta*.

The species of *O. uchidae* was classified within the genus *Okiscarta* [19], but in our studies was placed consistently in *Cosmoscarta* and grouped with *C. exultans*. Morphologically, *O. uchidae* was firstly placed in *Cosmoscarta* [18]. However, Matsumura [19] proposed that *Okiscarta* was closely allied to *Cosmoscarta*, but could be easily distinguished from the latter especially in the following characters: thorax posteriorly produced so as to conceal scutellum except for the apex; tegmina at the apices blunt, approximately twice as long as the pronotum; ovipositor on each side depressed deeply, much shorter than the pygophore. However, after comprehensive review of the type material of the Oriental cercopid spittlebug species, *Okiscarta* and *Cosmoscarta* were proposed as generic synonymies [20]. Furthermore, one Cercopoidea phylogenetic analysis based on DNA nucleotide sequence data from six loci demonstrated similar result that *O. uchidae* was placed within *Cosmoscarta*, despite having a low support value [21], providing molecular evidence for our placement of *Okiscarta* species within *Cosmoscarta*. Here we initially added support for this conclusion with the phylogenetic analyses of mitogenome data. Both the ML bootstrap and BI posterior probability presented strong signals and therefore highly supported the inclusion of *O. uchidae* within *Cosmoscarta*, which otherwise would be paraphyletic. This result was also supported by nucleotide similarity among species, the patterns of intergenic spacer *trnS2-nad1*, the models of substitution models observed in the secondary structures of tRNAs, and the tree topologies tests. These results support the necessity to revise the boundaries of this genus to generate a classification consistent with the evolutionary history of this group of spittlebugs. Given that mitogenomes are powerful molecular markers for inferring phylogenetic relationships at different taxonomic levels within Hemiptera [8, 33, 51–53], we concluded that the previously defined *Okiscarta* species was derived from *Cosmoscarta* and, therefore, here we transfer *O. uchidae* to *Cosmoscarta*.

Although our study provided the first mitochondrial phylogeny of Cosmoscartini, only two genera of *Ectemnonotum* and *Cosmoscarta* (if redefined to include *Okiscarta*) were available. Considering the limited representatives of mitogenomes in each Cosmoscartini genus, a denser taxon sampling from Cosmoscartini is still needed for further clarification of the placement of *Okiscarta* within *Cosmoscarta* and the placements of *Cosmoscarta* and *Ectemnonotum* within Cosmoscartini.

## Conclusions

Our study presents the mitogenomes of nine Cosmoscartini species and is the first representation of the detailed comparative genomic and phylogenetic analyses within Cercopidae. Newly sequenced Cosmoscartini mitogenomes share similar genomic features, but present high variation in control regions among the five complete mitogenomes. The evolutionary patterns of PCGs indicate that their evolution is under purifying selection, in which *atp8* and *cox1* exhibit the highest and lowest evolutionary rate, respectively. The tRNA secondary structures have diverse substitution patterns in Cosmoscartini, with each stem generally more conserved than the corresponding loop. The secondary structures of rRNAs are predicted for the first time within Cercopidae, with conserved regions distributed unevenly both in *rrnL* (domain IV and V) and *rrnS* (domain III). Cosmoscartini mitogenomes are punctuated by non-coding portions highly conserved in size, except for the spacers of *trnS2-nad1* and the control region. Among them, the longest spacer is the control region, which appears to be a fast-evolving sequence characterized by variable length of (15–355 bp) repeated motifs. However, the spacer of *trnS2-nad1* possesses a conserved motif, which may be the bidirectional transcription termination factor.

Although the status of *Okiscarta* is still under debate with regards to morphological characters, the previously defined genus *Okiscarta* is suggested to be a synonym of *Cosmoscarta* according to the mitogenome data. Within *Cosmoscarta*, the four closely related species sharing high similarity in morphology are also clearly resolved. Therefore, full mitogenomes provide a better understanding of phylogenetic relationships at a low taxonomic level (genus or species). This study has provided an initial taxonomic recommendation and might assist in future mapping of mitogenomic evolution and phylogenetic relationships in Cercopidae.

## Methods

### Samples and DNA extraction

Nine adults of Cosmoscartini species were collected from seven locations (Additional file 6: Table S4). All specimens were preserved in absolute ethyl alcohol and stored in − 20 °C freezer in Institute of Zoology, Chinese Academy of Sciences until use. Total genomic DNA was extracted from legs of a single sample using the DNeasy Blood & Tissue kit (Qiagen Hilden, Germany) following the manufacturer’s instructions.

### PCR amplification and sequencing

The mitogenomes of all species were generated by amplified overlapping fragments, using a set of newly designed primers (Additional file 7: Table S5). PCR amplification was performed using TaKaRa LA Taq (Takara Bio Inc., Dalian, China), with the following conditions: 2 min at 92 °C; 40 cycles of 30 s at 92 °C, 30 s at 48–55 °C, 12 min at 60 °C; and a final extension step at 60 °C for 20 min. PCR products were electrophoresed in a 1% agarose gel, purified, and sequenced directly or cloned into the pMD™19-T vector (Takara Bio Inc., Dalian, China). All fragments were sequenced on an ABI 3730XL DNA sequencer by Majorbio Biotechnology Company (Beijing, China) with primers walking on both strands.

### Bioinformatic analysis

Sequences were assembled using SeqMan program included in the Lasergene software package (DNAStar Inc., Madison, Wisc.). The tRNAs were identified by Mitos WebServer [54], with the Mito genetic code of invertebrate. PCGs, rRNAs and control regions were confirmed by the boundaries of tRNAs, and by alignment with other Cercopidae gene sequences. To ensure the accuracy of gene boundaries, PCGs were also translated into amino acids according to the invertebrate mitochondrial genetic code. The comparable sequence identity map was depicted by the CGView Comparison Tool [55], with query size of 50 bp in each BLAST search. Base composition, codon distribution, and relative synonymous codon usage (RSCU) were calculated by MEGA 6.05 [56]. Composition skew analysis was calculated according to the formulas: AT skew = (A-T)/(A + T) and GC skew = (G-C)/(G + C) [57]. The rate of synonymous substitutions (Ks), the rate of nonsynonymous substitutions (Ka), and the ratio of Ka/Ks were determined with DnaSP 5.0 [58]. Tandem repeats and stem-loop structures of control regions were identified by the tandem repeats finder online server [59] and the Mfold Web Server [60], respectively.

### Estimates of rRNA secondary structure

Secondary structures of rRNAs were inferred following the models proposed for some other hemipterans [33, 34, 42, 44]. Helix numbering was named according to the convention established at the Comparative RNA Web (CRW) Site [36]. Sequences lacking significant homology were also folded by the Mfold Web Server.

### Phylogeny

Phylogenetic analyses were performed on the nine newly sequenced mitogenomes of Cosmoscartini, with three other Cercopidae species used as outgroups (Additional file 6: Table S4). Nucleotide sequences for all the 13 PCGs were translated into amino acids, aligned using Muscle implemented within MEGA 6.05, and then toggled back into nucleotide alignments. The tRNAs and rRNAs were aligned with MAFFT 7.310 using the Q-INS-i algorithm [61]. Additionally, to eliminate poorly aligned positions and divergent regions, Gblocks 0.91b [62] was employed with default settings. Phylogenetic analyses were conducted with four datasets: (1) P123: all codon positions of 13 PCGs; (2) P12: first and second codon positions of PCGs; (3) P123R: P123, 22 tRNAs, and two rRNAs; and (4) P12R. Each combined dataset was concatenated by BioEdit 7.0.9.0 [63].

Maximum Likelihood (ML) analyses were conducted using IQ-TREE 1.6.5 [64] with 1000 replicates of ultrafast likelihood bootstrap [65]. Partitioned analyses [66] for multi-gene alignments were defined by both gene types (each of 13 PCGs, 22 tRNAs, and two rRNAs) and codon positions (first, second, and third codon positions for each PCG). The best-fit model for each partition was determined by ModelFinder [67] implemented in the IQ-TREE program.

Bayesian inference (BI) was performed with the site-heterogeneous model CAT + GTR implemented in PhyloBayes MPI 1.5a [68] through the online CIPRES Science gateway [69]. Two independent searches were run until the likelihood of the sampled trees stabilized and the two runs converged satisfactorily (maxdiff < 0.1). The initial 25% trees of each run were discarded as burn-in, and the consensus tree was computed from the remaining trees. The phylogenetic trees were visualized in FigTree 1.4.2 (http://tree.bio.ed.ac.uk/software/figtree/).

### Topology tests

To compare alternative phylogenetic hypotheses, ML trees constrained for the monophyly of *Cosmoscarta* were calculated with IQ-TREE 1.6.5 using the same search strategies described above. *P*-values were obtained for the Approximately Unbiased (AU) [70], Kishino-Hasegawa (KH) [71], and Shimodaira-Hasegawa (SH) [72] tests with IQ-TREE 1.6.5.

## Additional files

Additional file 1: **Table S1.** Gene content of our nine sequenced specimens and other published Cercopidae mitogenomes. (XLSX 24 kb)

Additional file 2: **Table S2.** Codons usage for the 13 protein-coding genes of nine Cosmoscatini species. (XLSX 24 kb)

Additional file 3: **Table S3.** Start and stop codons of 13 protein-coding genes of nine Cosmoscatini species. (XLSX 10 kb)

Additional file 4: **Figure S1.** Sequence alignments of the intergenic spacer between *trnS2* and *nad1* in the nine Cosmoscartini mitogenomes, with the conserved nucleotides marked with *. (TIF 162 kb)

Additional file 5: **Figure S2.** Organization of the control regions in the five complete Cosmoscartini mitogenomes. The tandem repeats are presented by colored oval with Arabic numerals inside. Non-repeat regions are shown by gray bars with sequence length inside. (TIF 3876 kb)

Additional file 6: **Table S4.** Samples used in this study, collecting data, and GenBank accession numbers. (XLSX 19 kb)

Additional file 7: **Table S5.** Primer used in this study. (XLSX 17 kb)

## Abbreviations

*atp6* and *atp8*: ATPase subunits 6 and 8
BI: Bayesian Inference
*cob*: Cytochrome b
*cox1-cox3*: Cytochrome c oxidase subunits I-III
mitogenome: Mitochondrial genome
ML: Maximum Likelihood
*nad1-nad6* and *nad4L*: NADH dehydrogenase subunits 1–6 and 4 L
PCG: Protein-coding gene
*rrnL* and *rrnS*: Large and small subunit ribosomal RNA (rRNA)
RSCU: Relative synonymous codon usage
tRNA: Transfer RNA
