# Supplementary figures and images for: Comparative analysis of the mitochondrial genomes of oriental spittlebug trible Cosmoscartini: insights into the relationships among closely related taxa
Source: BMC Genomics. 2018 Dec 27;19:961. doi: 10.1186/s12864-018-5365-7 (PMC6307326; doi:10.1186/s12864-018-5365-7)

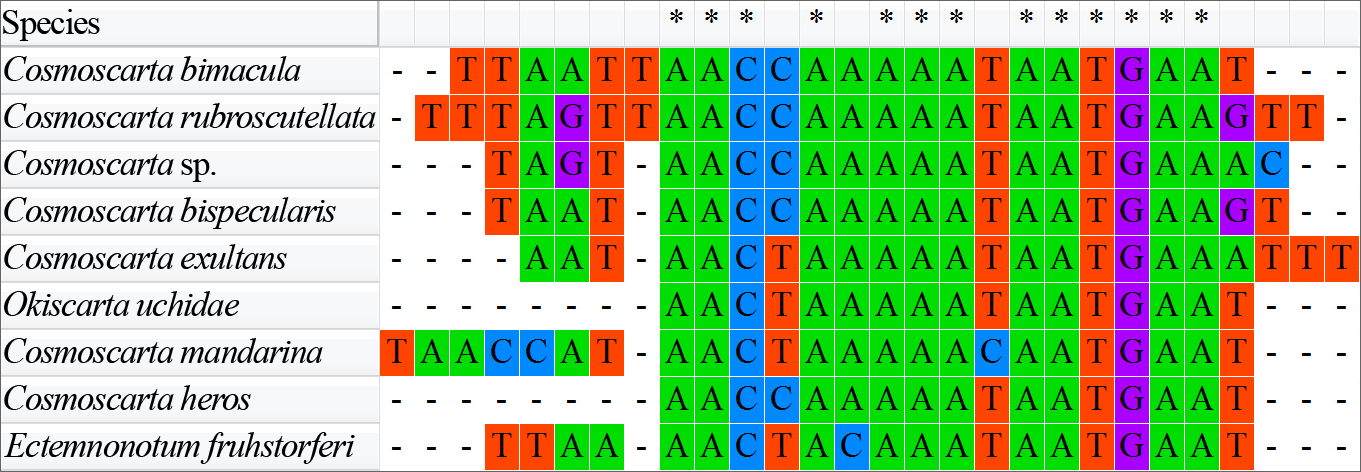

Supplement: Supplementary file 4 — Figure S1. Sequence alignments of the intergenic spacer between trnS2 and nad1 in the nine Cosmoscartini mitogenomes, with the conserved nucleotides marked with *. (TIF 162 kb) [file 12864_2018_5365_MOESM4_ESM.tif]
